# *Hibiscus sabdariffa* L. and Its Bioactive Constituents Exhibit Antiviral Activity against HSV-2 and Anti-Enzymatic Properties against Urease by an ESI-MS Based Assay

**DOI:** 10.3390/molecules22050722

**Published:** 2017-04-30

**Authors:** Sherif T. S. Hassan, Emil Švajdlenka, Kateřina Berchová-Bímová

**Affiliations:** 1 Department of Natural Drugs, Faculty of Pharmacy, University of Veterinary and Pharmaceutical Sciences Brno, Palackého tř. 1946/1, 612 42 Brno, Czech Republic; svajdlenkae@vfu.cz; 2 Department of Applied Ecology, Faculty of Environmental Sciences, Czech University of Life Sciences Prague, Kamýcká 129, 165 21 Praha-Suchdol, Czech Republic; berchova@knc.czu.cz

**Keywords:** anti-HSV-2 activity, ESI-mass spectrometry-based assay, urease inhibitors, protocatechuic acid, *Hibiscus sabdariffa* L., bacterial infection

## Abstract

For decades, *Hibiscus sabdariffa* L. and its phytochemicals have been shown to possess a wide range of pharmacologic properties. In this study, aqueous extract of *Hibiscus sabdariffa* (AEHS) and its bioactive constituent protocatechuic acid (PCA), have been evaluated in vitro for their antiviral activity against HSV-2 clinical isolates and anti-enzymatic activity against urease. Antiherpetic activity was evaluated by the titer reduction assay in infected Vero cells, and cytotoxicity was evaluated by the neutral red dye-uptake method. Anti-urease activity was determined by a developed Electrospray Ionization-Mass Spectrometry (ESI-MS)-based assay. PCA showed potent anti-HSV-2 activity compared with that of acyclovir, with EC_50_ values of 0.92 and 1.43 µg∙mL^−1^, respectively, and selectivity indices > 217 and > 140, respectively. For the first time, AEHS was shown to exert anti-urease inhibition activity, with an IC_50_ value of 82.4 µg∙mL^−1^. This, combined with its safety, could facilitate its use in practical applications as a natural urease inhibitor. Our results present *Hibiscus sabdariffa* L. and its bioactive compound PCA as potential therapeutic agents in the treatment of HSV-2 infection and the treatment of diseases caused by urease-producing bacteria.

## 1. Introduction

Herpes simplex virus (HSV) infections are quite common in humans, affecting about 90% of the world population. HSV is a member of *Herpesviridae*, a wide family of enveloped-DNA viruses that cause several clinically significant syndromes in both adults and neonates [1]. HSV-2 is mainly connected with genital infection, and has been recorded as a risk factor of HIV infection in humans [2]. Currently, treatment of HSV-2 infection mainly relies on the use of acyclovir and related nucleoside analogues that target viral DNA polymerase. Unfortunately, although several strategies have shown high efficacy results, HSV-2 infection treatment fails in about 30–45% of infected adults [3,4]. This is due to the extensive use of acyclovir and related nucleoside analogues, which has created drug resistance, associated with other adverse effects, alongside with the establishment of viral latency and reactivation that occurs in the presence of humoral- and cell-mediated immunity [5]. To date, no prophylactic HSV vaccine has been found to be entirely effective in the prevention of HSV infections [6,7]. Therefore, it is crucial to find alternative strategies to combat this viral resistance, and increase treatment efficacy results.

Urease (urea amidohydrolase, EC 3.5.1.5), is a nickel-containing metalloenzyme that catalyzes the hydrolysis of urea to ammonia and carbamate; the latter decomposes spontaneously to produce another molecule of ammonia and carbon dioxide, enhancing the rate of the uncatalyzed reaction by a factor of 8 × 10^17^ [8,9]. High concentrations of ammonia arising from this reaction, and the accompanying pH elevation, have important negative effects on humans and agriculture as well. Urease is widely distributed in Nature, including plants, fungi, algae, bacteria and even in the human gut and kidneys [10,11]. In recent years, urease has been considered a significant virulence factor implicated in infections of the urinary and gastrointestinal tracts [12]. In addition, urease is the main cause of pathologies induced by *Helicobacter pylori* as it allows the pathogen to survive at the low pH of the stomach and grow and multiply, spreading infection to the inner layers of gastroduodenal mucosa, resulting in producing gastritis and peptic ulceration, which in some cases may lead to cancer [13]. All these negative implications can be managed by inhibition of urease [14]. However, while urease inhibitors such as acetohydroxamic acid (AHA) and phosphoramidates have shown therapeutic efficacy, limitations associated with severe side effects, such as teratogenicity, psycho-neurological, and musculo-integumentary symptoms, have limited their use in the treatment of urinary and gastrointestinal tracts infections [10] Therefore, in recent years, the search for various groups of urease inhibitors with different types of inhibition, various mechanisms of action, and minimal side effects has gained much attention in the research field [15]. Natural products and their derivatives have long been used as a source of new drug candidates in drug discovery. This is due to their great diversity of the chemical structures and better drug-like properties of many of these molecules compared to synthetic compounds [16,17].

*Hibiscus sabdariffa* L. (*Malvaceae*; *H. sabdariffa*) is a plant mainly cultivated in tropical and subtropical regions with a long history of herbal and edible uses worldwide [18,19]. It is an annual or perennial plant or woody-based shrub with serrate leaves, red calyces and red stems. This plant has a long tradition of medicinal use as it contains a rich profile of bioactive compounds responsible for its therapeutic efficacy, including antimicrobial, antiparasitic, anticancer, and antiinflammatory properties [20,21]. Protocatechuic acid (PCA), an active compound in *H. sabdariffa*, has been also shown to exert various pharmacologic properties, including but not limited to antimicrobial and antioxidant activities [22]. This study was designed to evaluate in vitro antiviral activity of AEHS and PCA against HSV-2 clinical isolates and to evaluate their safety for topical administration as well as anti-urease activity of AEHS by a developed ESI-MS-based assay.

## 2. Results

### 2.1. Determinations of Concentration of PCA and Total Polyphenols Content in Plant Material

**Figure 1:**
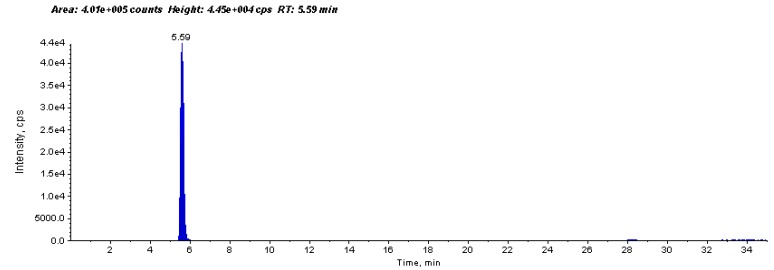
HPLC-MS chromatogram shows identification (two MRM transitions *m*/*z* 153→109 and 153→91) and determination of concentration of protocatechuic acid (PCA) in aqueous extract of *Hibiscus sabdariffa* (AEHS). PCA was detected at retention time (RT): 5.59 min and quantified using an external calibration method with standard PCA (94.1 µg/g dry weight of *Hibiscus* calyces).

The results demonstrated that the concentration of PCA in AEHS was found to be 94.1 µg/g dry weight of *Hibiscus* calyces (Figure 1) and total polyphenols content calculated as mg of gallic acid equivalent was 106.0 mg/g of dry extract of *Hibiscus* calyces.

### 2.2. Anti-HSV-2 Activity and Cytotoxicity

AEHS and PCA were evaluated with respect to their inhibitory effect on HSV-2 replication. Before performing the antiherpetic assay, we assessed the cytotoxicity of each sample in Vero cells by the neutral red dye-uptake method. The CC_50_ values for PCA and acyclovir were found to be higher than 200 µg∙mL^−1^ (Table 1). Antiherpetic activity was determined by the titer reduction assay in infected Vero cells using quantitative real-time reverse transcription PCR. Ten acyclovir-sensitive strains of HSV-2 (clinical isolates) were used and typed by quantitative real-time reverse transcription PCR using primers pairs H_2_M_40_ 5′-GTACAGACCTTCGGAGG-3′ and H_2_P_4_ 5′-CGCTTCATCATGG GC-3′ for identification. AEHS was not active against HSV-2. This could be related to the low concentrations of antiherpetic compounds in the crude extract. PCA showed potent anti-HSV-2 activity compared with that of acyclovir with EC_50_ values of 0.92 and 1.43 µg∙mL^−1^, respectively, and selectivity indices > 217 and > 140, respectively. PCA exhibited cytotoxic effect on Vero cells at concentration higher than its EC_50_. The selectivity index (SI) is fundamental to determine any possible toxic effect of any compound on the cells that could be confused with an antiviral activity. Based on our results, PCA demonstrated anti-HSV-2 activity with SI > 217.4 higher than acyclovir (> 140). Thus, the SI verifies the safety index of PCA.

### 2.3. Anti-Urease Inhibitory Properties by ESI-MS-Based Assay

Urease inhibitors play an essential role as alternative antibiotics in the treatment of gastrointestinal and urinary tract infections caused by urease-producing bacteria [8]. Enzyme activity and inhibition studies were determined by an ESI-MS based method. The method is based on the detection of urea depletion in the absence and presence of inhibitors by monitoring catalytic reaction through simultaneous detection of the concentration changes of urea.

#### 2.3.1. Determination of Urease Kinetic Parameters *K*_m_ and *V*_max_

**Figure 2:**
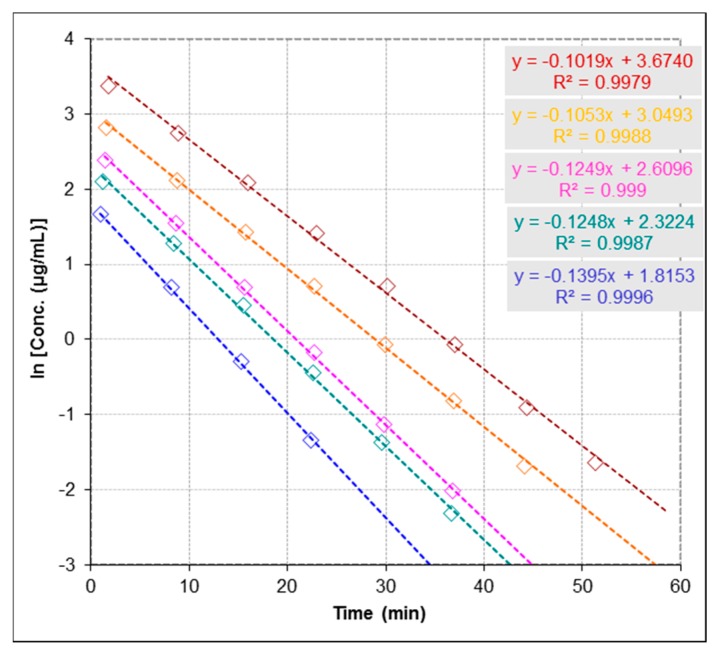
Urease-catalyzed reaction evaluated by Electrospray Ionization-Mass Spectrometry (ESI-MS) based assay at five concentrations of urea as shown in the figure 137.0 [●], 208.2 [●], 274.0 [●], 416.4 [●] and 694.1 [●] µmol∙L^−^^1^ and fixed concentration of urease 60.0 µg∙mL^−^^1^ by monitoring catalytic reaction through simultaneous detection of the concentration changes of urea, where the slopes represent the reaction rate constants (k_0_) in the absence of inhibitors. Concentrations changes of urea are presented as logarithms of concentration. The precision of time course analysis was calculated as RSD (%) of multiple measured slopes (lower than 10%). For clarity of figure, multiple measurements have not been presented.

**Figure 3:**
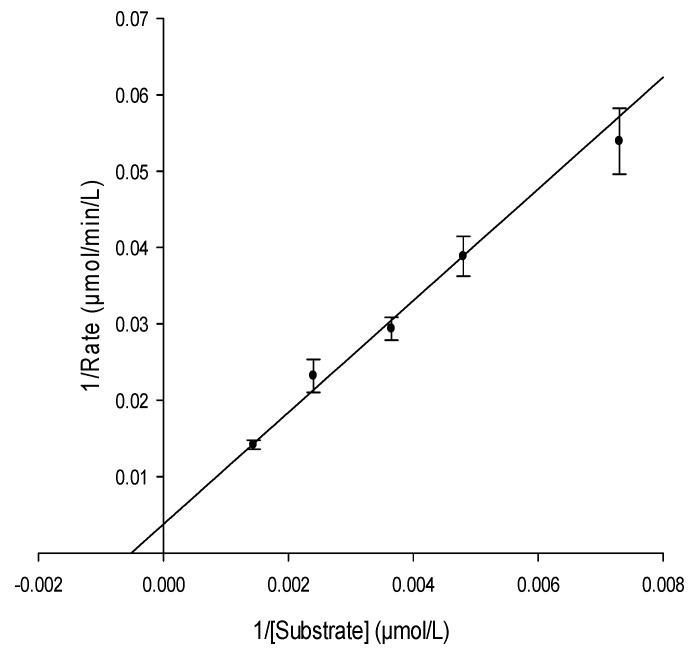
Lineweaver Burk plot for determination of urease kinetic parameters; Michaelis constant (*K*_m_) and the maximal reaction rate (*V*_max_). *K*_m_ = 1888.7 µmol∙L^−1^ and *V*_max_ = 259.6 µmol∙L^−1^∙min^−1^. *R*^2^ = 0.99.

It is known that the essential function of enzymes is to enhance the rate of biochemical reactions. Therefore, to understand enzyme function, it is very crucial to study the kinetic description of their activity. To determine kinetic parameters of the urease-catalyzed reaction including the Michaelis constant (*K*_m_) and the maximal reaction rate (*V*_max_), we monitored the enzymatic reaction through decreasing of concentration of urea at five concentrations (137.0, 208.2, 274.0, 416.4 and 694.1 µmol∙L^−1^) and constant concentration of urease (60.0 µg∙mL^−1^, Figure 2). The initial rate of the reaction was determined for each concentration from the rate constant and initial substrate concentration. A plot of 1/initial velocity versus 1/(urea) generates a Lineweaver-Burk plot and a linear regression fit to this data was used to determine the *K*_m_ and *V*_max_ (Figure 3). The good linearity of the Lineweaver-Burk plot (*R*^2^ = 0.99) for urea shown in Figure 3 ensures the accuracy of steady-state kinetics in this method. *K*_m_ of urease was determined to be 1888.7 µmol∙L^−1^ and *V*_max_ to be 259.6 µmol∙L^−1^∙min^−1^. Values obtained by the present method are in excellent agreement with the range of values reported in previous studies (1–4 mM for *K*_m_ and 0.0298–4.0 mM/min for *V*_max_) [23,24,25].

#### 2.3.2. Determination of Half Maximal Inhibitory Concentration (IC_50_)

**Figure 4:**
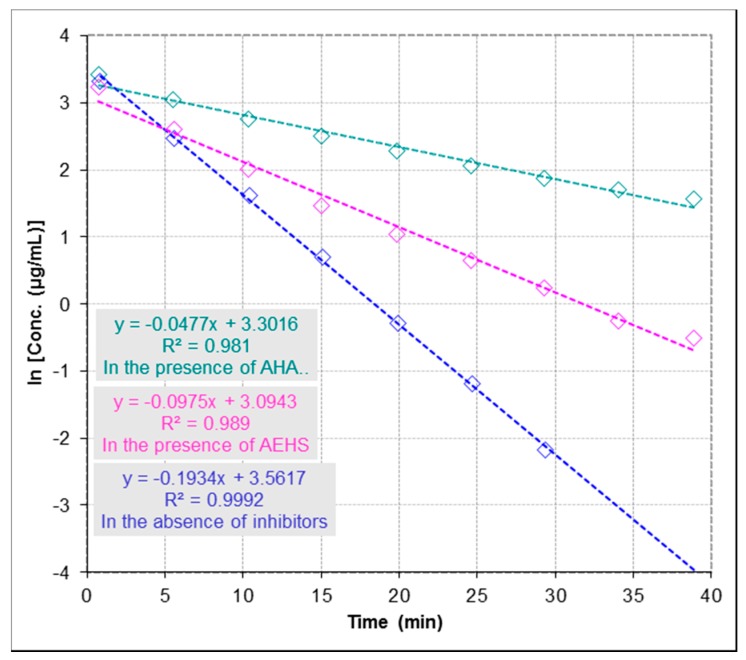
Effect of aqueous extract of *Hibiscus sabdariffa* (AEHS) and acetohydroxamic acid (AHA) on the inhibition of urease activity by Electrospray Ionization-Mass Spectrometry (ESI-MS) based assay. Urease activity and inhibitory properties of AHA and AEHS were assayed as described in Experimental section, where k is the reaction rate constant in the presence of AHA [●] and AEHS [●] (k = 0.0477 and 0.0975 min^−1^, respectively) and k_0_ is the reaction rate constant in the absence of inhibitors [●] (k_0_ = 0.1934 min^−1^). Concentrations changes of urea are presented as logarithms of concentration. IC_50_ for AEHS was determined to be 82.4 µg∙mL^−1^ and for AHA to be 4.3 µmol∙L^−1^. The precision of time course analysis was calculated as RSD (%) of multiple measured slopes (lower than 10%). For clarity of figure, multiple measurements have not been presented.

For inhibitor screening, the IC_50_ value is an apparent measure of the potency of an inhibitor at specific experimental conditions. Figure 4 shows the reaction progression slope for the depletion of urea in the presence and absence of inhibitors following the first-order kinetics conditions per equation C_t_ = C_0_ × e^−kt^, where C_t_ is the concentration at time (t), C_0_ is the initial concentration and k is the reaction rate constant [26]. From Figure 4, the reaction rate constant in the presence of inhibition (k) is lower than the reaction rate constant in the absence of inhibition (k_0_), where k in the presence of acetohydroxamic acid (AHA; used as a reference urease inhibitor) and AEHS (k = 0.0477 and 0.0975 min^−1^, respectively) and (k_0_ = 0.1934 min^−1^) at concentrations of 274.0 µmol∙L^−1^ for urea and 60.0 µg∙mL^−1^ for urease, and concentrations of inhibitors (AHA = 13.2 µmol∙L^−1^ and AEHS = 81.0 µg∙mL^−1^). It has been reported that the percent inhibition is defined as percent reduction of the reaction rate constant (k) as compared with the rate constant in the absence of an inhibitor (k_0_). Thus, % activity is usually determined as k/k_0_ and % Inhibition is 1 – % activity. Therefore, to determine IC_50_ value of an inhibitor the following equation % activity = k/k_0_ =1 − [I]/([I] + IC_50_) = IC_50_/([I] + IC_50_), where [I] is the concentration of the inhibitor, was used as previously described [26]. IC_50_ for AHA was determined to be 4.3 µmol∙L^−1^ and for AEHS to be 82.4 µg∙mL^−1^. The used method proved to be robust and determined IC_50_ value for AHA compared favourably with previous data reported in literature (IC_50_ for AHA = 5 µM) [27].

#### 2.3.3. Repeatability and Stability Studies

**Figure 5:**
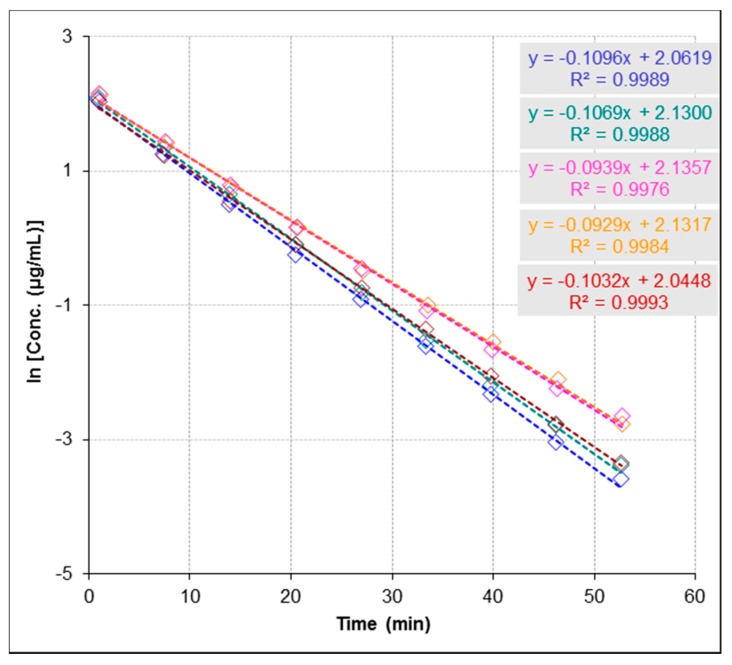
Determination of repeatability of measuring urease-catalyzed reaction evaluated by Electrospray Ionization-Mass Spectrometry (ESI-MS) based assay at fixed concentration of urea 416.4 µmol∙L^−1^ and fixed concentration of urease 60.0 µg∙mL^−1^ (repeated five times; as shown in different colors). Relative standard deviation (RSD) of slopes is 7.5%.

Precision of the method was verified by repeatability and stability studies. The measurements demonstrated a very good repeatability (Figure 5), where the relative standard deviation (RSD) was found to be 7.5%. Although current practices in stability studies rely on the addition of an internal standard to assure that short-term and long-term signal fluctuations do not influence quantitative analyses, we avoided the use of an internal standard which could interfere with enzymatic reaction or exact determination of substrate concentration. Therefore, we measured a calibration curve after each experiment to correct instability of MS signal.

## 3. Discussion

For decades, treatment of infectious diseases has been a focus of interest for both researchers and healthcare providers, as the arising issue of drug resistant strains has become a serious problem worldwide. This study adds to the growing body of knowledge on the antiviral activity of polyphenol-enriched extracts derived from plants. We show for the first time that PCA, an active compound of *H. sabdariffa* L. exerts potent antiviral activity against clinical isolates of HSV-2, the mechanism of which involves the inhibition of viral replication. Plant-derived phytochemicals, such as polyphenols, phenolics, terpenes, alkaloids and other substances have been reported to possess inhibitory properties on HSV replication [7,28]. For instance, polyphenols have been found to interfere with the early phases of the HSV replicative cycle and/or with viral particles directly [29]. In this study, PCA showed excellent ability to inhibit HSV-2 replication and hence might open new gates for the development of anti-HSV-2 drugs. In addition, PCA is known to exhibit cytotoxic effect on cancer cells with various mechanisms of action [30,31]. Therefore, it should be taken into consideration the non-toxic doses, especially with topical preparations containing PCA. Considering the significant global incidence, morbidity, and mortality rates of viral sexually transmitted infections (STIs), the development of new, safe, topically applied antiviral agents for their prevention is of high priority [32]. Therefore, in recent years, a new approach has been under focus of interest to maximize the treatment efficacy by combining natural products with nucleoside analogues, resulting in reduction of cytotoxicity [33]. Thus, this approach will reduce the cytotoxicity of PCA. However, further studies should be carried out to determine its safety as well as the possibility of adverse effects in vivo.

Urease inhibitors are considered promising therapeutic agents for the treatment of ureolytic bacterial infections [34]. Over the past two decades, a large number of plant-derived products were found to possess in vitro inhibitory activities against urease and intensive efforts were then made to evaluate the efficacy of these inhibitors in vivo [35]. Unfortunately, most of these investigations failed to prove the efficacy of those studied drugs in vivo due to problems of hydrolytic instability, toxicity and adverse side effects [36]. In this study, we report for the first time the anti-urease activity of AEHS by a developed ESI-MS based assay. Since *H. sabdariffa* L. is commonly used in traditional folk medicine in the form of a herbal tea or as a dietary supplement [37]. This indicates that AEHS could be used safely as natural urease inhibitor for the treatment of diseases caused by urease-producing bacteria. Additionally, the use of AEHS could overcome the problem of preventing the use of urease inhibitors in vivo and in clinical trials; due to their possible toxicity, instability and undesirable side effects. It has been reported that polyphenols and phenolic compounds have been shown to possess potent anti-urease activity. Moreover, crude plant extracts containing polyphenols have been shown an excellent ability to interact with wide range of enzymes [8,12]. Therefore, we may suggest that anti-urease activity of AEHS could be related to its rich content of polyphenols. Identification of bioactive molecules from AEHS and confirmation of the key components contributing to anti-urease activity should be studied in further investigations.

## 4. Materials and Methods

### 4.1. Plant Collection and Extraction Procedure

*Hibiscus sabdariffa* L. was collected from the northern part of Aswan, Egypt in June 2014, and identified by Sherif T. S. Hassan in the Department of Natural Drugs, Faculty of Pharmacy, University of Veterinary and Pharmaceutical Sciences (Brno, Czech Republic). A voucher specimen of the plant was deposited with the number EGHS5 at the herbarium of the department. One-gram air dried *Hibiscus* calyces were extracted in 10 mL of boiled water by sonication extraction for 30 min. The extract was stored at 4 °C for further use. A 1 mL of aliquot of crude extract was evaporated to dryness and then weighted to yield a final amount of 0.0243 g of dry extract.

#### 4.1.1. Determination of Concentration of PCA in Plant Material

Air dried *Hibiscus* calyces (0.51 g) were extracted in 10 mL of boiled water by sonication extraction for 30 min. HPLC-MS (Agilent 1200 HPLC system, Böblingen, Germany) coupled with a mass spectrometer (Sciex-3200QTRAP-hybrid triple quadrupole/linear ion trap, Toronto, ON, Canada) fitted with Electrospray Ionization (ESI) were used for the analysis. For the quantification, an external calibration method with standard PCA (Sigma Aldrich, Prague, Czech Republic) was used as previously described [38].

#### 4.1.2. Determination of Total Polyphenols by Folin-Ciocalteu Method

The total polyphenols content was determined per the Folin-Ciocalteu method as previously described by ISO 14502-1 [39].

### 4.2. Antiviral Activity against HSV-2

#### 4.2.1. Viral Strains, Cultures, Cell Lines and Reagents

For antiviral activity, Vero cells (ATCC: CCL 81, UK; were obtained from the Motol University Hospital, Prague, Czech Republic) were grown in Eagle’s minimum essential medium (MEM; Cultilab, Campinas, UK) supplemented with 10% fetal bovine serum (FBS; Gibco, Carlsbad, UK), 100 U/mL penicillin G, 100 µg∙mL^−1^ streptomycin and 25 µg∙mL^−1^ amphotericin B (Sigma-Aldrich, Berlin, Germany) and maintained at 37 °C in 5% CO_2_ atmosphere. Ten clinical isolates of HSV-2 (isolated from patients with HSV-2 infection) were kindly obtained from the Motol University Hospital, Prague, Czech Republic). All clinical isolates were typed by quantitative real-time reverse transcription PCR using primers pairs H_2_M_40_ 5′-GTACAGACCTTCGGAGG-3′ and H_2_P_4_ 5′-CGCTTC ATCATGGGC-3′ for identification as previously described [40] and then were propagated in Vero cells. The cytopathic end-point assay was used to determine the titers which were expressed as 50% tissue culture infective dose (TCID_50_/mL) as previously described [41]. Viral stocks were stored at −80 °C.

#### 4.2.2. Determination of Cytotoxicity

Cytotoxicity was evaluated by the neutral red dye-uptake method as previously described [42]. Briefly, AEHS, protocatechuic acid (PCA; Sigma Aldrich, Prague, Czech Republic) and acyclovir (positive control; Sigma-Aldrich, Berlin, Germany) were prepared in 0.1% dimethyl sulfoxide (DMSO). Stock solutions were prepared in deionized water at concentration of 400 µg∙mL^−1^ and sterilized. Vero cell monolayers were cultivated in 96-well microtiter plates with two-fold serial dilutions of AEHS, PCA and acyclovir, and incubated for 48 h at 37 °C in 5% CO_2_ atmosphere. After incubation, the morphological alterations of the treated cells were determined using an inverted optical microscope (Leitz, Berlin, Germany) and the maximum non-toxic concentrations (MNTC) were determined. The 50% cytotoxic concentrations (CC_50_) of AEHS, PCA and acyclovir were calculated as the concentration that reduces cell viability by 50%, when compared to the untreated controls as priviously described [43].

#### 4.2.3. Anti-HSV-2 Activity

Antiherpetic activity was determined by the titer reduction assay as previously described [44]. Briefly, acyclovir was used as a positive control and Vero cell monolayers were treated with AEHS, PCA and acyclovir at concentrations at which no change was observed in cell morphology, and 80% cell viability were determined. 100 TCID_50_/mL of HSV-2 acyclovir-sensitive suspensions were added to treated and untreated cell cultures and incubated at 37 °C for 48 h in a 5% CO_2_ atmosphere. After incubation, the virus titers in treated and untreated cells, were determined. Anti-HSV-2 activity was evaluated as percentage inhibition (PI) using antilogarithmic TCID_50_ values as follows: PI = [1 − (antilogarithmic test value/antilogarithmic control value)] × 100. The dose response curve was determined from the MNTC, and the 50% effective concentration (EC_50_) was determined as the concentration required for 50% protection against virus-induced cytopathic effects. Selectivity index (SI) value was calculated as the ratio of CC_50_/EC_50_.

#### 4.2.4. Statistical Analysis

Experiments were generated in duplicate in at least three independent experiments. PRISM software version 5.0 (GraphPad Software, Inc., La Jolla, CA, USA) was used for statistical analysis (one-way ANOVA test) and to calculate EC_50_ and CC_50_ parameters.

### 4.3. Anti-urease Inhibitory Properties

#### 4.3.1. Enzyme, Substrate, Inhibitors and Reagents

All chemical reagents were obtained from commercial suppliers and used without further purification. Urease (Type III, from *Canavalia ensiformis*), urea, acetohydroxamic acid (AHA; standard urease inhibitor) were purchased from (Sigma Aldrich, Prague, Czech Republic).

#### 4.3.2. Instrumentation

**Figure 6:**
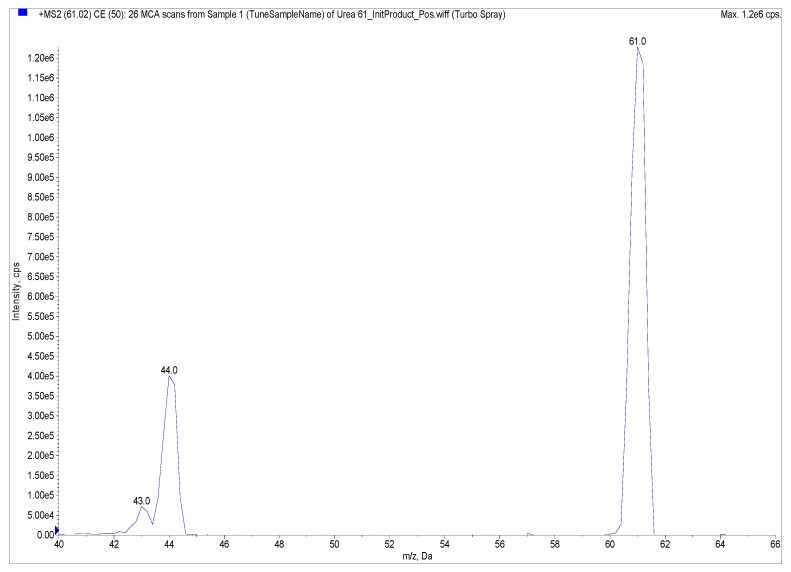
Multiple reaction monitoring (MRM) analysis for detection and quantitation of urea by triple quadrupole mass spectrometry coupled with Electrospray Ionization (ESI) in positive ion mode with transition *m*/*z* (61→44).

Urease inhibitory activity was evaluated using a system pump-injector (Agilent 1200) coupled with a Sciex-3200QTRAP- hybrid triple quadrupole/linear ion trap mass spectrometer fitted with Electrospray Ionization (ESI). The system runs in flow injection analysis (FIA) mode without a HPLC column. The operational parameter settings were as follows: curtain gas (CUR), 25 psi; nebulizer gas (GS1), 50; auxiliary gas (GS2), 40; declustering Potential (DP), 15 V; ion spray voltage, −4000 V; turbo temperature (TEM), 450 °C. MS in positive ion mode was used in multiple reaction monitoring (MRM) analysis for detection and quantitation of urea with transition *m*/*z* 61→44 (Figure 6). 0.1% HCOOH and 1 mM HCOONH_4_ were used as mobile phases with the flow rate set at 0.5 mL∙min^−1^. The injection volume was 10 µL.

#### 4.3.3. Anti-urease Activity by ESI-MS-Based Assay

Several important experimental conditions were investigated and taken into consideration to optimize the efficacy of the ESI-MS-based assay analysis, such as the buffer concentration, the buffer pH, and the type of sample vials. Under the optimized experimental conditions, experiments were employed and aimed at evaluating urease assay conditions that would be compatible with ESI-MS. The enzymatic reaction took place in 1 mM HCOONH_4_ buffer, which was found to be suitable (pH 7.5) containing 60.0 µg∙mL^−1^ of urease, over a substrate (urea) concentrations ranging from 137.0 to 694.1 µmol∙L^−1^ at room temperature (25 °C). Urea was chosen as a natural substrate for the enzymatic reaction. Urea concentrations were maintained at concentrations below 694.1 µmol∙L^−1^ to avoid excess substrate inhibition. The concentrations of inhibitors (AHA = 13.2 µmol∙L^−1^ and AEHS = 81 µg∙mL^−1^) were used and were within the range of their effectiveness to inhibit urease activity. Enzymatic reaction was carried out by pre-incubating urease in 1 mM HCOONH_4_ buffer with each inhibitor during 180 min to reach binding equilibrium followed by adding urea. The solutions were directly injected automatically into FIA system and the concentration changes of urea were monitored. For the analysis of the kinetics of substrate depletion by ESI-MS, areas (total counts) under peaks for substrate in the FIA record were integrated. Each measured sequence consists of five measurements. Briefly, to determine the repeatability of measurements, we performed multiple measurements of enzymatic reaction of the same sample. The precision of time course analysis was calculated as RSD (%) of multiple measured slopes (lower than 10%). The slopes represent rate constants, which are used for determination of enzyme activity and inhibition studies. For clarity of figures, multiple measurements have not been presented in Figure 2 and Figure 4. As shown in Figure 5, multiple measurements and evaluation of slopes are presented.

For measuring the rate constants for determining *K*_m_ and *V*_max_, we set to each experiment different substrate concentrations. Furthermore, for screening inhibitors, we set to each experiment different inhibitor concentrations or different types of inhibitor. A calibration curve after each experiment was measured to correct instability of MS signal.

## 5. Conclusions

Plant-derived phytochemicals provide an excellent option for the treatment of infectious diseases. The present findings indicated that PCA exhibited notable inhibitory properties on HSV-2 replication and thus has promising application in the development of anti-HSV-2 drugs. However, further studies should be carried out to evaluate its safety, validate the activity in vivo, and to eliminate the possible adverse effects of this compound by using improved delivery techniques prior its possible practical application. In addition, our results indicate that AEHS exerted anti-urease activity according to a developed ESI-MS-based assay. However, further studies must be performed to identify the active compounds in AEHS that contribute to the anti-urease activity, and evaluate their toxicity and structure-activity relationships.

## Figures and Tables

**Table 1:** Anti-HSV-2 activity and cytotoxicity of PCA and AEHS.

Compound	EC_50_ (µg∙mL^−1^)	CC_50_ (µg∙mL^−1^)	SI
AEHS	n.d	n.d	n.d
PCA	0.92 ± 0.21	>200	>217
Acyclovir	1.43 ± 0.43	>200	>140

All data were average values from three independent experiments; Values presented are means ± S.D. of three independent experiments performed in duplicate; CC_50_: 50% cytotoxic concentration; EC_50_: 50% Effective concentration; SI: Selectivity index (CC_50_/EC_50_); n.d: Not determined; PCA: Protocatechuic acid; AEHS: Aqueous extract of *Hibiscus sabdariffa*.
